# Panniculitis and vitiligo occurring during BRAF and MEK inhibitors combination in advanced melanoma patients: Potential predictive role of treatment efficacy

**DOI:** 10.1371/journal.pone.0214884

**Published:** 2019-04-02

**Authors:** Francesca Consoli, Ausilia Maria Manganoni, Salvatore Grisanti, Fausto Petrelli, Marina Venturini, Giovanni Rangoni, Francesco Guarneri, Paolo Incardona, William Vermi, Pier Giacomo Calzavara Pinton, Alfredo Berruti

**Affiliations:** 1 Department of Medical and Surgical Specialties, Radiological Sciences, and Public Health, Medical Oncology, University of Brescia at ASST-Spedali Civili, Brescia, Italy; 2 Department of Dermatology, University of Brescia at ASST-Spedali Civili, Brescia, Italy; 3 Oncology Unit, ASST Bergamo Ovest, Treviglio, Italy; 4 Department of Pharmacy, ASST-Spedali Civili, Brescia, Italy; 5 Department of Laboratory Medicine, Unit of Pathology, ASST-Spedali Civili, Brescia, Italy; 6 Department of Molecular and Translational Medicine, Section of Pathology, School of Medicine, University of Brescia at ASST-Spedali Civili, Brescia, Italy; Universidade de Sao Paulo, BRAZIL

## Abstract

Panniculitis and vitiligo-like lesions have been recently identified as rare cutaneous side effects of the combination of BRAF and MEK inhibitors, a standard of care in metastatic and locally advanced BRAF V600 mutated melanoma. An immune-mediated mechanism has been advocated in the pathogenesis of these skin lesions. Herein we retrospectively reviewed our institutional experience with the aim to explore the association between the occurrence of panniculitis and vitiligo-like lesions during combination therapy with dabrafenib (D) and trametinib (T) and outcome of advanced melanoma patients. Among 52 consecutive BRAF V600 mutated melanoma patients submitted to DT in our center, 12 (23%) developed immune related skin lesions (IRSLs): 8 panniculitis and 4 vitiligo. Patients with IRSLs diagnosis obtained a better disease response (83% versus 25%) (p = 0.001) than their counterpart and had a longer progression free survival and overall survival. The association of IRSLs and lower risk of disease progression (HR 0.19; CI 95% 0.04–0.90; p = 0.043) was confirmed after adjusting for major prognostic factors in multivariate analysis. IRSLs might represent an easy predictive surrogate marker for treatment response and favourable outcome in melanoma patients submitted to DT combination therapy.

## Introduction

The combination of BRAF and MEK inhibitors, such as dabrafenib (D) and trametinib (T), actually represents the standard of care in metastatic or locally advanced BRAF V600 mutated malignant melanomas (MM) [1,2].

The results of pooled analyses of phase II and III studies revealed that DT combination leads to a long-term benefit in terms of either progression-free survival (PFS) (48% and 38% of patients free from progression at 1 and 2-years respectively), or overall survival (OS) (74% and 53% of patients alive after 1 and 2-years, respectively). The survival obtained by DT was superior to that of single agent BRAF inhibitor D, either in the overall population or in patient subsets stratified according to commonly recognized prognostic characteristics [3]. Baseline serum lactate dehydrogenase (LDH) and number of metastatic sites strongly predicts OS and PFS of DT treated patients [4] and patients with normal LDH and less than three metastatic sites are expected to have a 5-years OS rate of 51% [5]. These findings notwithstanding, the prediction of prognosis of individual melanoma patients treated with DT remains difficult and new prognostic markers are needed, in addition beside BRAF mutation, no additional predictive parameters of DT efficacy have been up to now identified.

Dermatologic complications, such as rash, dry skin, photosensitivity, squamo-proliferative growth, hand-foot skin reactions have been frequently observed during the administration of BRAF and MEK inhibitors [6]. More recently, two additional cutaneous side effects have been described: panniculitis and vitiligo-like lesions [7,8,9]. The pathogenesis of these cutaneous reactions is not fully elucidated, although an immune-mediated mechanism is advocated [10]. Panniculitis was interpreted as a part of a systemic non-infectious inflammatory reaction, while drug-induced vitiligo as an immune-mediated destruction of melanocytes. Panniculitis during combination therapy was reportedly rare [7, 11]. Vitiligo-like lesions were observed during BRAF inhibitor mono-therapy but they were rarely reported during the combination of BRAF and MEK inhibitors [8,9,12].

Preclinical and clinical evidences provide support for an immune-modulating effect of target therapies, through the enhancement of a favorable tumor microenvironment. BRAF inhibition could enhance melanoma antigen expression and facilitate T-cell cytotoxicity [13]. Recent data are in favour of a pre-existing tumor immunity features associated to response to target therapies and the identification of specific gene signature suggests a correlation with an immune response to treatments [14,15]. Indeed, the immune system activation could account for the long-term disease control observed in a subset of patients with the combination of dabrafenib and trametinib [2,4,5,16].

A recently published retrospective series showed a significant association between immune adverse reactions including vitiligo, erythema nodosum, uveitis and keratitis sicca and durable response to BRAF inhibitors either administered alone or in association with MEK inhibitors [12].

In this paper, we report a case series of patients who developed immune related skin lesions (IRSLs) during DT therapy with the aim to correlate these events with disease response and patient progression-free survival (PFS).

## Patients and methods

From February 2015 to September 2018, we retrospectively collected data from 52 consecutive treatment naïve patients with advanced BRAF V600 mutated metastatic melanoma, who received dabrafenib (150 mg twice daily) and trametinib (2 mg daily) combination therapy, at the Medical Oncology Unit of University of Brescia at Spedali Civili in Brescia (Italy). Patients were evaluated monthly to assess safety and efficacy of treatment and a full skin examination was performed at each visit.

The diagnosis of panniculitis or vitiligo-like lesions was documented clinically and the diagnosis was confirmed by a dermatologist, actively involved during the diagnostic workflow. A cutaneous biopsy was performed only in some cases to confirm doubtful patterns of presentation. A biochemical assessment was conducted at the time of the diagnosis of panniculitis, including complete blood count, erythrocyte sedimentation rate and antistreptolysin O (ASO) titers and C-reactive protein (CRP), to exclude alternative etiologies.

This observational study was conducted in accordance with the good clinical practice guidelines and was approved by the Independent Ethic Committee of the ASST-Spedali Civili in Brescia, Italy.

We explored the associations between baseline clinical and biochemical characteristics (such as number and type of metastatic sites, level of LDH, Performance Status ECOG) and post-baseline characteristics (such as panniculitis or vilitigo-like lesions secondary to treatment exposure) with the Chi-square test or the Fisher test as required. To assess the impact of clinical factors in predicting overall response rate (ORR) a binary logistic regression was used both in univariate and multivariate analysis. For survival outcomes, PFS curves stratified by single covariates were generated with the Kaplan-Meier method and the difference between curves was assessed with the log-rank test. The prognostic value of clinical parameters on PFS was evaluated with the Cox proportional hazard model in uni- and multivariate backward conditional analysis. All tests were two-sided and the statistical significance was set at p <0.05. Hazard ratio for PFS from the present and other published studies were pooled according to fixed or random effect model according to heterogeneity. Higgins’ I^2^ statistic values < and >50 were defined as having low and high heterogeneity, respectively. For all other analyses the SPSS software version 23.0 (SPSS Inc, Chicago, IL; USA) was used. The data of the present series and the published one [12] were aggregated into a meta-analysis by RevMan 5.3 software (Cochrane Collaboration, Copenhagen, Denmark) and Comprehensive Meta-analysis Software 3.

## Results

### Patients’ characteristics and treatments

Demography and patient characteristics were depicted in Table 1. All patients received DT combination. Twelve patients (23%) underwent immune related skin lesions (IRSLs): 7 females and 1 male had panniculitis and 3 male and 1 female had vitiligo-like dermatitis.

**Table 1:** Baseline characteristics and demographics of the entire population of fifty-two patients.

Total patients	N = 52 (%)
**Age (years)**	
Median (range)	56 (28–77)
**Sex**	
Male	34 (65)
Female	18 (35)
**Braf mutation**	
V600E	45 (86)
V600K	4 (8)
Others V600	3 (6)
**Metastatic melanoma stage**	
M1a	7 (14)
M1b	11 (21)
M1c	34 (65)
**Brain metastases**	
Yes	18 (35)
No	34 (65)
**LDH**	
Normal	39 (63)
Increased	13 (37)
**Number of metastatic site**	
< 3	29 (56)
≥ 3	23 (44)
ECOG PS*	
0–1	33 (63)
≥ 2	19 (37)

* ECOG PS = Eastern Cooperative Oncology Group Performance Status.

**Fig 1:**
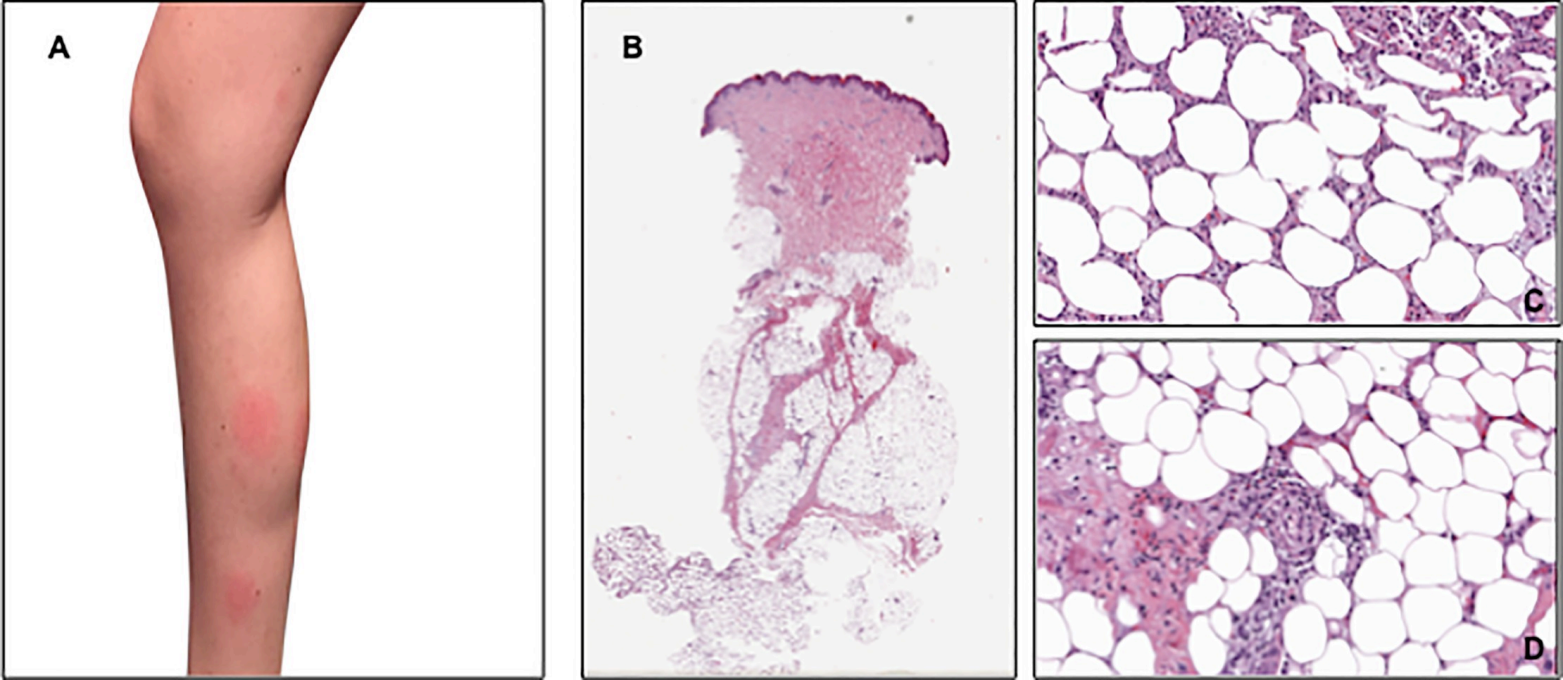
Clinical presentation of the skin eruptions and histopathological findings in one patient. (A) shows the clinical lesions at the left leg. (B and C) show histological findings consistent with neutrophilic lobular panniculitis, without vasculitis (D).

The median time to first occurrence of panniculitis was 8 months (range 1–16). This condition appeared during the first month of treatment in 2 cases, while a late onset (i.e. after at least 7 months from the beginning of therapy) was observed in 6 patients. Legs were involved in all cases (Fig 1A), while in three cases, the disease also appeared on the arms. Skin lesions’ biopsies, performed in three patients, documented the presence of lobular or septal panniculitis without vasculitis (Fig 1B, Fig 1C, Fig 1D).

The eruptions had a self-limiting control and typically resolved within few days or weeks, but recurrence occurred in all cases due the continuing treatment. Dermatitis was accompanied by systemic symptoms, such as fever and/or arthralgia, in all cases. No history of chronic concomitant diseases was documented and no additional medications were introduced. Complete blood count, erythrocyte sedimentation rate were normal and ASO titers resulted negative, while CRP resulted increased during the concomitance with systemic symptoms. Patients were treated symptomatically with non-steroidal anti-inflammatory drugs (NSAID), in association with topical or systemic steroids with good symptomatic clinical benefit. No treatment discontinuation was required due to IRSLs onset.

The median time to first onset of vitiligo-like lesions was 16 months (range 6–40 months). Vitiligo-like lesions had an atypical and patchy distribution of the depigmented macules, they occurred initially on the trunk in all patients. The depigmented areas progressed during time, involving head and upper limbs in two patients and also lower limbs in one of them. Confocal microscopy evaluation of vitiligo areas showed the loss of dermal papillary ring brightness and some «half-rings» structures with «scalloped border-like» features and a homogeneous reduction of the normal brightly refractile papillary rings at dermo-epidermal junction.

Patients destined to develop IRSLs had at baseline conditions lower tumor burden (less than three metastatic sites) and normal level of LDH in comparison to their counterpart (p = 0.007 and p = 0.024 respectively, at Fisher’s two tailed test).

### Relationship between the occurrence of IRSLs and progression-free survival (PFS)

At univariate analysis, variables that were found to be significantly associated to PFS were LDH (normal versus elevated) (HR 0.16; Confidence Interval [95%CI] 0.07–0.38, p<0.0005), IRSLs (presence versus absence) (HR 0.13; CI 95% 0.03–0.56, p = 0.006), number of metastatic sites (< 3 versus ≥ 3) (HR 0.36; CI 95% 0.17–0.78; p = 0.009) and M stage (M1a and M1b versus M1c according to American Joint Committee Cancer system 7^th^ edition) (HR 0.33; CI 95% 0.13–0.82, p = 0.017) (Table 2).

**Table 2:** Univariate e multivariate analysis of factors associated to progression-free survival (PFS) in BRAF mutated melanoma patients treated with dabrafenib and trametinib.

Covariate	Effect tested	PFS (months)			Univariate analysis			Multivariate analysis		
					PFS			PFS		
		Median	95% CI	p	HR	95% CI	P	HR	95% CI	P
**Metastatic sites**	< 3	32	17.61–46.38	0.005	0.36	0.17–0.78	**0.009**	0.74	0.32–1.70	0.482
	≥ 3	8	3.51–12.48							
**M stage**	M1a, M1b	32	12.64–51.36	0.010	0.33	0.13–0.82	**0.017**	0.53	0.18–1.56	0.256
	M1c	8	5–11							
**LDH**	Normal	22	8.93–35.07	<0.0005	0.16	0.07–0.38	**<0.0005**	0.30	0.12–0.75	**0.010**
	Elevated	5	2.70–7.29							
**IRSLs**	IRSL+	n.r.		0.001	0.13	0.03–0.56	**0.006**	0.19	0.04–0.90	**0.043**
	IRSL-	9	4.64–13.35							

n.r. not reached; PFS Progression-free survival; HR hazard ratio; CI confidence interval; IRSLs+ presence of immune related skin lesions; IRSLs- absence of immune related skin lesions

**Fig 2:**
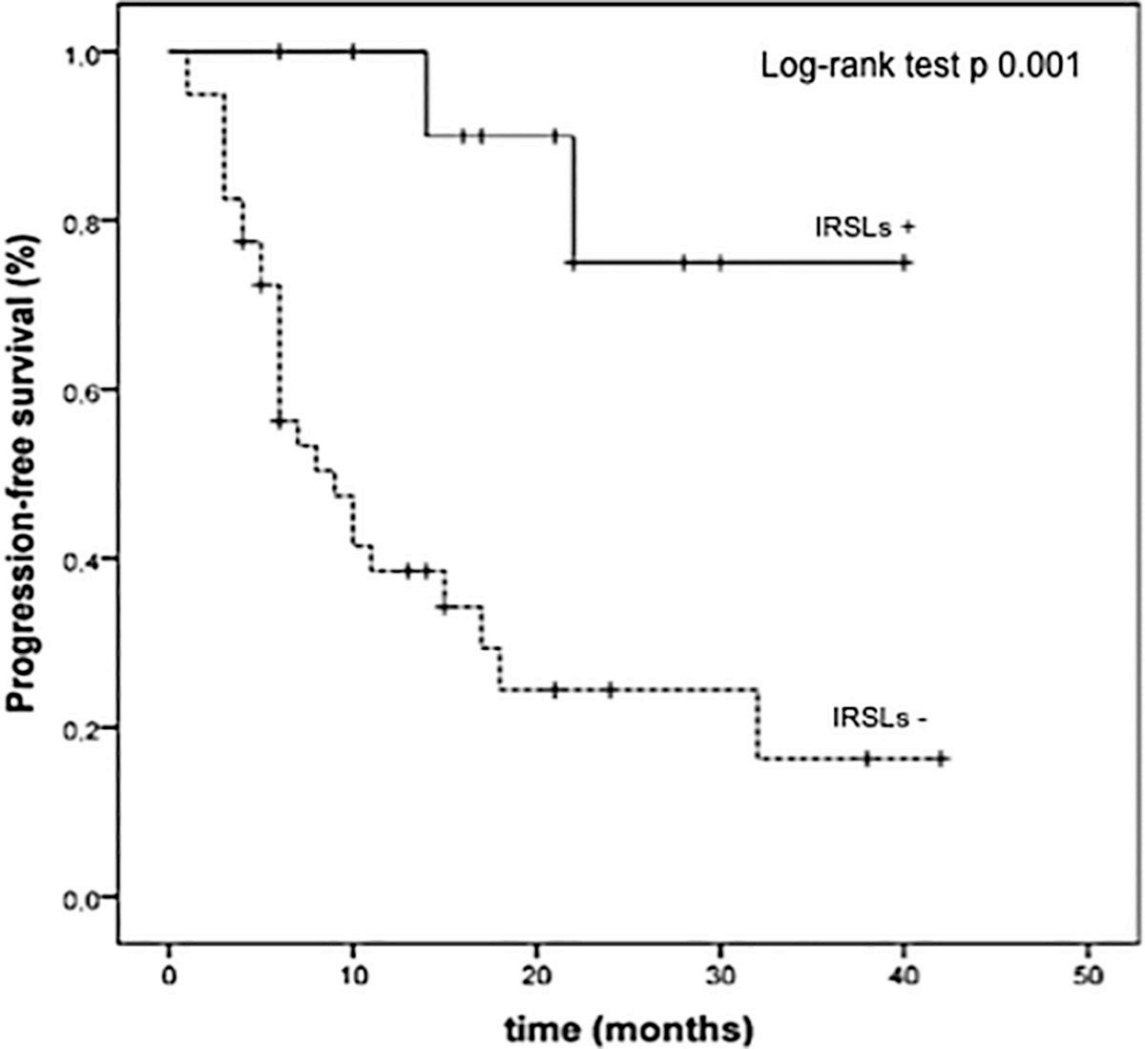
Kaplan Meier estimates of progression-free survival (PFS) in patients with (IRSLs+) or without immune relate skin lesions (IRSLs-).

**Fig 3:**
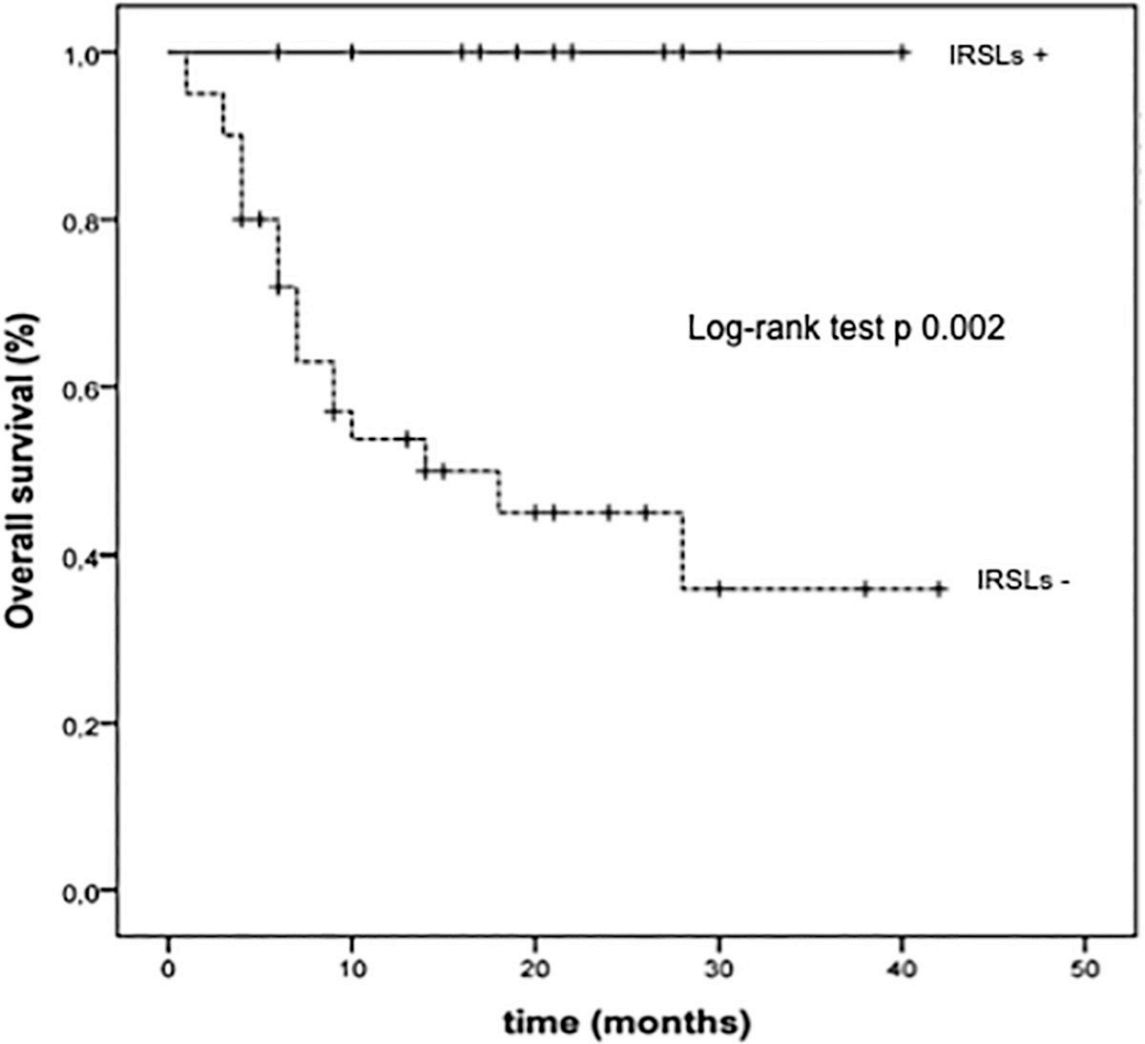
Kaplan Meier estimates of overall survival (OS) in patients with (IRSLs+) or without immune relate skin lesions (IRSLs-).

At the last follow-up examination, 2 out of 12 patients (17%) experiencing IRSLs underwent disease progression after DT therapy and all of them were alive. The corresponding proportions of patients who did not develop IRSLs were 27 out 40 with disease progression (67%) and 20 out of 40 alive (50%), respectively. The Kaplan Meier curves showed that the median PFS was not reached versus 9 months (95%CI 4.64–13.35 months) (p = 0.001) and the median OS was not reached versus 13 months (p = 0.002) in patients experiencing IRSLs or not, respectively (Figs 2 and 3).

At multivariate analysis, normal LDH value at baseline and the occurrence of IRSLs were the only 2 variables independently associated with a lower risk of disease progression (p = 0.010 and p = 0.043, respectively) (Table 2).

Since there were no deaths in the IRSLs group, the univariate and multivariate Cox analysis for overall survival was not performed.

**Fig 4:**
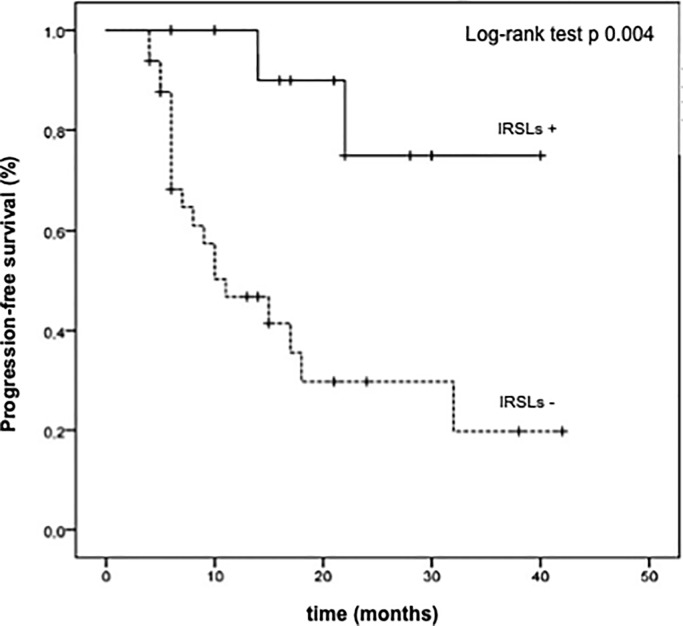
Landmark analysis of progression-free survival (PFS) at 3 months in patients with (IRSL+) or without immune related skin lesions (IRSL-).

The longer PFS in the subgroup of patients experiencing IRSLs maintained the statistical significance even after introducing a landmark of 3 months to prevent the bias of early progression (median PFS not reached versus 11 months in the patient subgroup with or without IRSLs, respectively) (p = 0.004) (Fig 4).

### Relationship between the occurrence of IRSLs and disease response to therapy

In the present series DT combination confirmed to be an active scheme since, according to RECIST criteria version 1.1, 8 patients (15%) obtained a complete response (CR), 12 patients (23%) a partial response (PR), 3 patients (6%) disease stabilization while 29 patients (56%) progressed. The overall response rate (CR+PR) was 38%. Ten out of 12 patients (83%) developing IRSLs attained a disease response to DT treatment; the corresponding response rate in patients without IRSLs was 25% (10 out 40 patients). This difference in response rate attained the statistical significance (Chi-square test, p = 0.001). No patient destined to develop IRSLs had a primarily resistant disease to DT. Two patients experienced disease progression after an initial response: one with panniculitis discontinued treatment after complete remission and progressed after 9 months; the second, with vitiligo-like lesions, had cerebral disease progression after a partial response lasting 17 months. Both of them were crossed to an anti-PD1 therapy and were alive at the last follow-up. The multivariate logistic regression analysis revealed that LDH level and IRSLs occurrence were the only significant independent predictors of clinical response (Table 3).

**Table 3:** Univariate and multivariate regression analysis of factors associated to response to target therapy.

Covariate	Effect tested	Responses			Univariate regression analysis			Multivariate regression analysis		
					Clinical Benefit (CR+PR+SD)			Clinical Benefit (CR+PR+SD)		
		CR+PR	SD	PD	HR	95% CI	P	HR	95%CI	P
**Metastatic sites**	< 3	16	3	11	0.17	0.04–0.58	**0.005**	0.44	0.10–1.95	0.28
	≥ 3	4		18						
**M stage**	M1a, M1b	10	2	6	0.23	0.07–0.80	**0.021**	0.56	0.12–2.4	0.442
	M1c	10	1	23						
**LDH**	Normal	19	3	17	0.06	0.008–0.54	**0.012**	0.10	0.01–0.91	**0.042**
	Elevated	1		12						
**IRSLs**	IRSL+	10		2	0.09	0.01–0.50	**0.006**	0.16	0.02–0.87	**0.034**
	IRSL-	10	3	27						

CR complete response; PR partial response; SD stable disease; PD progressive disease; HR hazard ratio; CI confidence interval; IRSLs+ presence of immune related skin lesions; IRSL- absence of immune related skin lesions

### Pooled analysis of immune-related adverse events (irAEs) during BRAF target therapies and melanoma patients’ outcome

**Fig 5:**
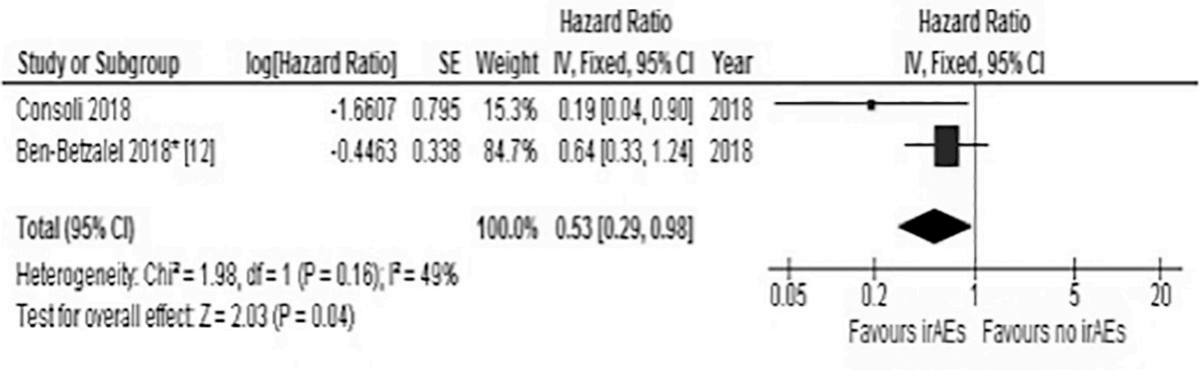
Meta-analysis of effect of immune related adverse effect during target therapy on progression-free survival (PFS). * Calculated Hazard Ratio and Confidence interval (CI) from the survival curves provided in the original report of Ben-Betzalel et al [12]. irAEs, immune related adverse events.

Our data and those published by Ben-Betzalel et al [12] were evaluated in a pooled analysis. As depicted in Fig 5, the pooled HR of the 2 studies (HR 0.53, CI 95% 0.29–0.98, p = 0.04) was significantly in favour of the association of immune related adverse events (irAEs) onset with a lower risk of progression.

## Discussion

The use of combination regimens with BRAF and MEK inhibitors has contributed to modify the prognosis of patients with BRAF V600 mutated melanomas and preclinical and clinical evidences actually available, suggest that the long term efficacy of these therapies could be attributed in part to an immune-modulating effect [16]. Prognostic parameters assessed at baseline conditions, such as tumor burden, levels of LDH and Performance Status have been repeatedly found to correlate with the disease response and patient prognosis [3,4,5].

In this report, we performed a retrospective analysis on 52 consecutive patients who were homogeneously treated with BRAF and MEK inhibitors, as current standard treatment in BRAF mutant metastatic disease. We provided explorative information on whether immune related skin lesions, occurring during combined target therapy, could be associated with a durable response of melanoma lesions and improve the patient outcome. Interestingly, patients experiencing IRSLs obtained a consistently higher response rate than patients who did not (83% versus 25%). Moreover PFS significantly favored patients with IRSLs, after adjusting for major prognostic factors (HR 0.19, p = 0.043).

Our data are consistent to those recently published by Ben-Betzalel et al [12] who explored the role of immune related adverse events (irAEs), as clinical predictor of response to treatment in 78 patients submitted to BRAF targeting therapies. They defined as immune related events vitiligo, erythema nodosum, uveitis and keratitis sicca and the median time to occurrence of irAEs from the therapy start was 6.3 months. The results of this retrospective analysis revealed an association between irAEs and PFS after adjustment for LDH and number of metastatic sites. The pooled analysis of our paper to that of Ben-Betzalel et al confirmed the PFS advantage associated with the occurrence of IRSLs.

The patient population of the Ben-Betzalel et al study, however, was different from that presented in this paper, since it consisted mainly of patients treated with BRAF inhibitor monotherapy, that was adopted in the past when the combination treatment was not the standard of care. Moreover, the frequency of erythema-nodosum like lesions in this paper (defined as red tender cutaneous nodules), that are compatible with the diagnosis of panniculitis, was significantly lower than that observed in our patients. In addition, the reported ocular side effects were not observed in our population. Panniculits is notoriously a short lived dermatitis, so the diagnosis may escape in the absence of repeated careful skin examinations by expert dermatologists during therapy. The greater proportion of panniculitis in our patients is probably due to the strict integration between oncologists and dermatologists both being involved at every time visit in each DT treated patients. Moreover, we noticed a rapid involution of individual painful inflammatory nodules and plaque without scarring or identifying the presence of a contusion appearance.

The correlation between immune-related side effects and both disease response and patient prognosis suggests a synergic interplay between the cytotoxic effect of BRAF and MEK inhibitors combination and immune system activation. Molecular target therapy could counteract the immunosuppressive effect of oncogenic BRAF signaling, leading to beneficial local and systemic immune-modulating effects [17]. Along this line, irAEs or IRSLs could be considered an *in-vivo* manifestation of immune system activation and the late onset of these side effects underlined the latency of immune system activation. Noteworthy, in this study panniculitis was detected earlier than vitiligo and this underlines the importance of the careful diagnosis this skin abnormality.

The retrospective nature, the limited number of patients included and the relatively short follow-up are the most important limitations of this study. Our data need confirmation in a larger patient series recruited prospectively.

In conclusions, the identification of surrogate parameters of efficacy of modern therapies in advanced melanoma is of paramount importance. The immune related skin lesions, panniculitis and vitiligo, occurring during treatment with BRAF and MEK inhibitors, are easily detectable and contribute to identify a patient subset destined to obtain a long-term clinical benefit from the therapy. The surrogacy role of these parameters deserves validation.
